# Femoral fracture classification in the Swedish Fracture Register – a validity study

**DOI:** 10.1186/s12891-019-2579-z

**Published:** 2019-05-08

**Authors:** Sara Brandt Knutsson, David Wennergren, Alicja Bojan, Jan Ekelund, Michael Möller

**Affiliations:** 1 Institute of Clinical Sciences, Sahlgrenska Academy, University of Gothenburg, Gothenburg, Sweden; 2 Department of Orthopaedics, Sahlgrenska University Hospital Gothenburg, SE-413 45 Gothenburg, Mölndal, Sweden; 3 Center of Registers, Western Healthcare Region, SE-413 45 Gothenburg, Sweden

**Keywords:** Fracture classification, Validity, Fracture register, Femoral fractures

## Abstract

**Background:**

A total of more than 270,000 fractures are registered in the Swedish Fracture Register (SFR), a national quality register. Fractures are classified following the AO/OTA classification, commonly by a junior doctor. As a step in the process of validating the data in the SFR, several studies of the accuracy of the fracture classification have already been published. The aim of this study was to evaluate the accuracy of femoral fracture classification in the SFR.

**Methods:**

One hundred and eighteen femur fractures were randomly selected from the SFR. Three experienced orthopaedic surgeons individually classified these fractures on two separate occasions and a gold standard classification was established. This classification was compared with the original classification in the SFR. Inter- and intraobserver agreement was calculated.

**Results:**

The agreement between the classification in the SFR and the gold standard classification was kappa = 0.65 for the AO/OTA group and kappa = 0.83 for the AO/OTA type. This corresponds to substantial and almost perfect agreement, according to Landis and Koch. The kappa values for interobserver agreement ranged from 0.64–0.76 for the AO/OTA group and 0.76–0.85 for the AO/OTA type. The kappa values for intraobserver agreement ranged from 0.79–0.81 for the AO/OTA group and 0.91–0.93 for the AO/OTA type.

**Conclusions:**

The classification of femoral fractures in the Swedish Fracture Register is substantial (AO/OTA group) to almost perfect (AO/OTA type) and as accurate as in previous studies. The present study also shows that the agreement between the SFR classification and a gold standard classification is in the same range of agreement as between experienced raters. In contrast to previous studies, the classifications in the SFR are made by an unselected group of mostly inexperienced classifiers. The results indicate that the fracture classification in a national quality register can be accurate enough to permit the evaluation of fracture treatment in specific groups of fractures.

## Background

In the Swedish Fracture Register (SFR), all fracture types in adults and all long-bone fractures in children are registered. The SFR is a unique national quality register, as it contains information on fractures, regardless of treatment; surgical or non-surgical. Subsequent treatments are included and the main outcome parameters are re-operation frequency and patient-reported outcomes. Seventy-five per cent of the hospitals in Sweden that treat fractures on a regular basis participate. A total of over 270,000 fractures have been registered since the register was started in 2011 [1].

Since 2012, all femoral fractures treated at Sahlgrenska University Hospital have been registered in the SFR. Each fracture is classified according to the AO/OTA classification. The registration and thereby the classification is most often made by the attending physician at the accident and emergency department (A&E).

The data in the SFR have to be proven valid in order to be useful. One important step in the process of validating the data is to determine the accuracy of the fracture classification.

Three previous studies of the accuracy of the AO/OTA classification in the SFR have been published. Wennergren et al. showed that, in the SFR, there was substantial agreement for fracture type (3 signs) and moderate agreement for fracture group (4 signs) in both tibial and humeral fractures [2, 3]. Juto et al. showed that there was substantial agreement for both fracture type and group in ankle fractures [4]. Previous studies of the accuracy of femoral fracture classification reach the same level of agreement, but the data are more variable [5–8] .

The aim of this study was to evaluate the accuracy of the fracture classification of femoral fractures in the SFR. Accuracy was defined as the level of agreement between the AO/OTA classification in the SFR and a gold standard classification. Another aim was to evaluate inter- and intraobserver agreement.

## Methods

### Classification of fractures

The primary registration of a fracture in the SFR is made by the physician who diagnoses the fracture, commonly a junior orthopaedic resident on call. The registration consists of the mechanism of injury, ICD code, AO/OTA fracture classification and, if appropriate, more specific data on open fracture, periprosthetic fracture, atypical fracture and so on. After surgical treatment, the operating surgeon will subsequently complete the registration regarding the type of surgical treatment given, including the type of implant. The surgeon can also re-classify the fracture if he or she doesn’t agree with the original classification.

No specific training in fracture classification is given, but the classification of fractures is part of the general training of orthopaedic residents.

The registration and classification process is performed online. The segment of the femur (proximal, diaphyseal or distal) is chosen. In each segment, there are nine different options of AO/OTA pictograms for fracture group. Short fracture descriptions are available for each picture.

To evaluate the accuracy of the classification of fractures, the true classification needs to be defined, i.e. the gold standard classification. In the present study, we have used the method described by Wennergren et al., which is based on the recommendations described by Audigé et al. [2, 9] The gold standard classification is defined as three experienced orthopaedic surgeons agreeing on the classification of a fracture. This gold standard classification is compared with the actual classification in the SFR.

Interobserver agreement is defined as the level of agreement between two different raters, whereas intraobserver agreement is the level of agreement between the same rater on two occasions.

### Data collection

The registration of femoral fractures in the SFR began in 2012. Between 1/1/2014 and 31/12/2014, 1202 femoral fractures were registered at Sahlgrenska University Hospital. Of these, 129 fractures were randomly selected. All radiographs available at the primary registration were collected by one of the authors (SB).

**Fig. 1:**
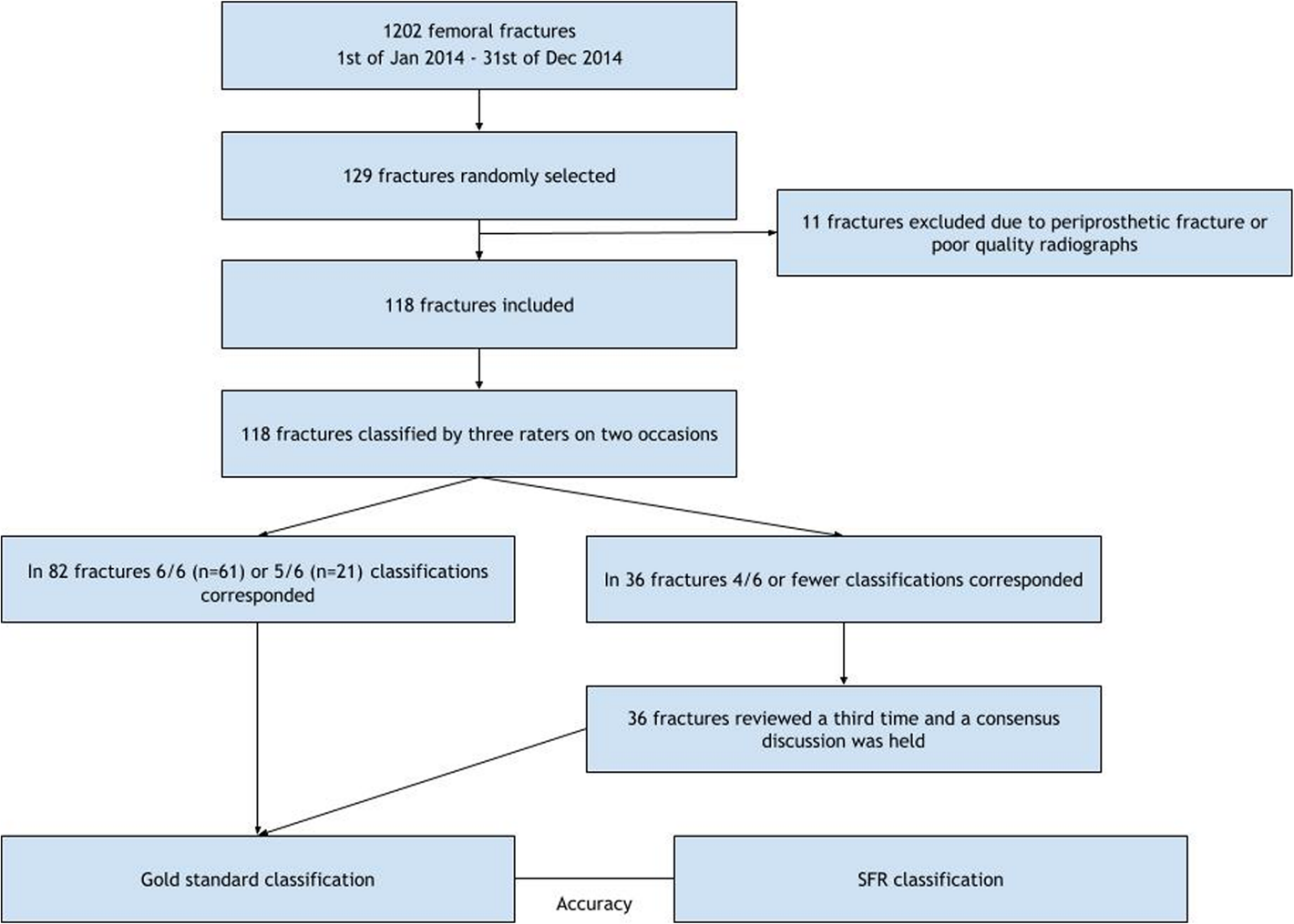
Flow chart of the study design

The radiographs were de-identified. Periprosthetic fractures were excluded. The radiographs considered to be of too poor quality, for example, if there was only one projection, were also excluded. A total of 11 radiographs were excluded. The final number of fractures available for evaluation was 118 (Fig. 1). Plain radiographs included antero-posterior and lateral views. In fractures where CT images were available at the primary registration, these scans were also made available to the three raters. Nineteen of the 118 patients underwent a CT scan prior to surgery.

### Measuring agreements

The expert group consisted of three experienced orthopaedic surgeons (MM, AB, DW), all well acquainted with the AO/OTA classification system. The de-identified radiographs were shown to the expert group at two classification seminars with a one-month interval. To mimic the conditions at registration in the SFR, pictograms of the different femoral fracture groups, together with a short description, were available. The three raters classified each fracture independently and without any clinical information. They were not allowed to see or discuss each other’s classification. At the second seminar, the procedure was repeated with the radiographs shown in a different order.

Each fracture was thus classified six times. When the classification corresponded in five or six of six times, this was considered the gold standard classification. When the classification was consistent in four or fewer of six times, the fracture was discussed at a third seminar. These radiographs were again shown to the raters de-identified and without any clinical information. The raters classified each fracture independently and were then presented with their previous classification(s). A discussion followed to reach consensus. As a result, gold standard classification was established for all 118 fractures.

### Statistics

Sample size calculations were performed to determine the number of fractures needed in the present study. Assuming kappa of approximately 0.5 and a relative error of 5%, a sample size of 116 fractures was required to obtain a width of the 95% confidence interval for kappa of 0.2. A loss of 10% was estimated and 129 fractures were therefore seleted from the SFR.

Cohen’s kappa is the statistical method which is often used to evaluate agreement between raters. In contrast to the percentage of agreement, kappa takes account of the possibility that the agreement may occur by chance [10].

Kappa is to be interpreted as follows, according to the guidelines of Landis and Koch [11] (Table 1).

**Table 1:** Interpretation of kappa

Kappa statistic	Strength of agreement
< 0	Poor
0–0.20	Slight
0.21–0.40	Fair
0.41–0.60	Moderate
0.61–0.80	Substantial
0.81–1.00	Almost perfect

The statistical analysis was made using SAS v 9.4. SAS Institute Inc., Cary, NC, USA.

### Ethics

The study was approved by The Regional ethical review board in Gothenburg. Reference number 174–16.

## Results

### Demographics

The total of 118 fractures consisted of 72 proximal (AO/OTA 31), 30 diaphyseal (AO/OTA 32) and 15 distal (AO/OTA 33) femur fractures, according to the established gold standard classification (Table 2). The raters classified one fracture as a pathological fracture, making the total number of fractures 117 in Tables 2 and 3. There were 77 women and 41 men. Their median age was 81 years, ranging from 17 to 98. The median age for women was 83 years, ranging from 19 to 98. The median age for men was 74 years, ranging from 17 to 95 (Table 3).

**Table 2:** Number of fractures for each fracture class according to the gold standard classification

	AO/OTA type				
AO/OTA group		1	2	3	Total
	31A	10	17	7	34
	31B	9	1	28	38
	31C	0	0	0	0
	32A	12	3	2	17
	32B	3	4	3	10
	32C	0	0	3	3
	33A	3	1	2	6
	33B	1	2	0	3
	33C	2	1	3	6

The raters classified one fracture as a pathological fracture, making the total number of fractures 117 in Table 2

**Table 3:** Distribution of patients as defined by the gold standard classification

Median age (range)	Women	Men	All
All fractures	83 (19–98); *n* = 77	74 (17–95); *n* = 40	81 (17–98); *n* = 117
Proximal femoral fractures (AO/OTA 31)	85 (49–98); *n* = 46	81 (33–95); *n* = 26	83 (33–98); *n* = 72
Diaphyseal femoral fractures (AO/OTA 32)	89 (19–94); *n* = 20	46 (17–79); *n* = 10	74 (17–94); *n* = 30
Distal femoral fractures (AO/OTA 33)	71 (51–88); *n* = 11	61 (29–68); *n* = 4	66 (29–88); *n* = 15

The raters classified one fracture as a pathological fracture, making the total number of fractures 117 in Table 3

### Classification

After two classification seminars, five or six classifications corresponded in 82 fractures (Fig. 1). This was regarded as the gold standard classification. For the remaining 36 fractures, each was shown a third time and a consensus discussion was held to define the gold standard.

The percentage of agreement comparing SFR classification with the gold standard classification for the AO/OTA group (4 signs) and type (3 signs) was 68 and 86% respectively (Table 4). Kappa was 0.65 for the AO/OTA group, which corresponds to substantial agreement according to Landis and Koch. For the AO/OTA type, kappa was 0.83, i.e. almost perfect agreement.

**Table 4:** Accuracy, defined as classification in the SFR compared with the established gold standard classification

	Accuracy	
	SFR vs GS	
	PA	Kappa (95% CI)
AO/OTA group (4 signs)	68%	0.65 (0.56–0.73)
AO/OTA type (3 signs)	86%	0.83 (0.75–0.9)

*PA* percentage of agreement, *GS* gold standard

Interobserver agreement ranged from kappa coefficients of 0.64–0.76 for the AO/OTA group and 0.76–0.85 for the AO/OTA type (Table 5).

**Table 5:** Interobserver kappa values with 95% confidence interval comparing the raters at the two classification seminars

	Interobserver agreement					
	Rater 1 vs Rater 2		Rater 1 vs Rater 3		Rater 2 vs Rater 3	
	Seminar 1	Seminar 2	Seminar 1	Seminar 2	Seminar 1	Seminar 2
AO/OTA group (4 signs)	0.64 (0.55–0.73)	0.72 (0.64–0.81)	0.76 (0.68–0.84)	0.73 (0.64–0.81)	0.65 (0.56–0.74)	0.69 (0.60–0.78)
AO/OTA type (3 signs)	0.76 (0.68–0.85)	0.83 (0.75–0.90)	0.85 (0.78–0.92)	0.81 (0.73–0.89)	0.78 (0.69–0.86)	0.80 (0.72–0.88)

Intraobserver agreement ranged from kappa coefficients of 0.79–0.81 for the AO/OTA group and 0.91–0.93 for the AO/OTA type (Table 6).

**Table 6:** Intraobserver agreement for each of the three raters, comparing their classification at the two seminars

	Intraobserver agreement					
	Rater 1		Rater 2		Rater 3	
	PA	Kappa (95% CI)	PA	Kappa 95% CI	PA	Kappa 95% CI
AO/OTA group (4 signs)	83%	0.81 (0.73–0.88)	81%	0.79 (0.72–0.87)	82%	0.8 (0.72–0.88)
AO/OTA type (3 signs)	95%	0.93 (0.88–0.98)	93%	0.91 (0.86–0.97)	93%	0.91 (0.86–0.97)

*PA* percentage of agreement, *GS* gold standard

## Discussion

The agreement on the AO/OTA classification of femoral fractures in the SFR compared with a gold standard classification was substantial (AO/OTA group) to almost perfect (AO/OTA type). An important part of the validity check of a fracture register is the accuracy of its fracture classification. The present study indicates that the classification of femoral fractures in the SFR is accurate and that the data can be reliably used for further research studies.

The interobserver agreement was substantial for the AO/OTA group and almost perfect for the AO/OTA type, as was the intraobserver agreement. This is in accordance with previous studies [5–8]. This shows that the agreement between the actual SFR classification and a gold standard classification is in the same range of agreement as it is between experienced raters.

Previous studies have focused on the classification of one segment of the femur, most often proximal femoral fractures [6–8]. In the present study, fractures of the entire femur were included. This makes the classification more challenging. Of the 118 fractures included, the classification in the SFR and the gold standard classification were identical in 68%, i.e. 80 fractures. In 11 of the remaining 38 fractures where classification in the SFR did not match the gold standard classification, there was a misclassification of the femoral segment. The most common difficulty was distinguishing a fracture in the distal part of the femoral shaft (AO/OTA 32) from a fracture in the proximal part of the distal femur (AO/OTA 33). Interestingly, even the expert raters had difficulty with the segments. Of the 11 fractures, ten were discussed at the raters’ third seminar, meaning that their original classification of each of these ten fractures was consistent in four or fewer of six times.

The results in the present study are equal to or better than those in previous studies. Meling et al. [5] evaluated 368 fractures of all femoral segments using the AO/OTA classification. This was part of a larger study examining 949 long-bone fractures registered in the Fracture and Dislocation Registry (FDR) at Stavanger University Hospital, Norway. A reference classification was defined and compared with the original classification in the FDR. The kappa values ranged from 0.33 for the AO/OTA type for femoral diaphyseal fractures to 0.9 for the AO/OTA type for proximal femoral fractures. Pervez et al. [8] evaluated the classifications of five raters with different levels of expertise in a study of 88 trochanteric hip fractures. The fractures were classified on two occasions, three months apart. The kappa values of interobserver agreement for the AO/OTA group ranged from 0.14–0.48. For the AO/OTA type, the kappa values ranged from 0.50–0.71.

The better results in the present study probably have multiple explanations. The use of the SFR in clinical practice makes the orthopaedic surgeons and residents more experienced with the AO/OTA classification of fractures. The online registration and classification process is pedagogically designed and easy to use, with pictograms for each fracture group. We also believe that the accuracy will be higher when validating fractures that are treated surgically, mainly due to a higher level of expertise in the classifying surgeon. In the present study, the effect could be of less importance, as many of the operations were performed by residents, the same individuals that are also in charge at A&E.

Among the 118 fractures, 19 underwent a CT scan prior to surgery. For these fractures, the percentage of agreement between the SFR classification and the gold standard classification was 52% for the AO/OTA group and 68% for the AO/OTA type. This is lower than the percentage of agreement for all fractures. It remains to be studied whether this difference is due to the fact that these fractures are actually harder to classify or whether there could be other reasons, for example, more information to consider.

Three similar studies of the validity of the classification of fractures in the SFR have been published. They all show results similar to those in the present study. Wennergren et al. [2] studied the accuracy of the classification of 114 tibial fractures in the SFR. The kappa values comparing the SFR classification with the gold standard classification were 0.56 for the AO/OTA group and 0.75 for the AO/OTA type. Juto et al. [4] studied the accuracy of the classification of 152 ankle fractures (AO/OTA 44) in the SFR. The kappa values were 0.67 for AO/OTA group and 0.77 for the AO/OTA type. Wennergren et al. [3] studied the accuracy of fracture classification of 116 humeral fractures in the SFR. The kappa values were 0.57 for the AO/OTA group and 0.66 for the AO/OTA type.

The question of whose classification we are validating can be raised when evaluating data in a national quality register like the SFR. The accuracy of the fracture classification in the present study is slightly higher than that of the three previous studies of the SFR. Femoral fractures are treated surgically to a larger extent than most other fractures. The classification could therefore have been made by the operating surgeon and not, as assumed, by the resident at A&E. In the present study of 118 fractures, 58 fractures were classified by the surgeon who performed the surgical procedure and 42 were primarily classified at A&E. In 11 of these 42 fractures, a re-classification had been made after the initial classification upon registration. The remaining 18 fractures were classified by a third person, which is the case with fractures in the SFR when neither the physician at A&E nor the surgeon registers the fracture. In contrast to many of the previous studies of femoral fracture classification, this study evaluates the classification of fractures in real life, i.e. upon registration in the SFR in everyday clinical practice. The aim of this study was to evaluate this process of registration and classification in a quality register made by a large group of physicians.

The finding that the agreement on AO/OTA classification in the SFR is equal to that in previous studies does not necessarily mean that it is sufficient. For the AO/OTA group (4 signs), the percentage of agreement was 68% (kappa = 0.65), meaning that the fracture classification in the SFR is incorrect on one of three occasions. It is possible to argue that this is not good enough to be able to use the data for research studies. Nevertheless, the SFR is much more useful for fracture evaluation than the standard registration, which is only ICD-10 code. Previous studies also show that the percentage of inter- and intraobserver agreement for fracture classification rarely exceeds 60–80% [12].

Expectations for the near future include the application of artificial intelligence in fracture classification. Olczak et al. recently published the first study of deep learning for skeletal radiographs. Five different deep learning networks were adapted for 256,000 fractures, meaning that the networks were trained to recognise the presence of a fracture. All the networks were benchmarked against a gold standard for 400 of the fractures. The percentage of agreement between the best performing network and the gold standard was kappa = 0.7 (83%). The network performed at the same level as two senior orthopaedic surgeons [13].

## Conclusions

The classification of femoral fractures in the Swedish Fracture Register is substantial (AO/OTA group) to almost perfect (AO/OTA type) and as accurate as in previous studies. The present study also shows that the agreement between the SFR classification and a gold standard classification is in the same range of agreement as between experienced raters. In contrast to previous studies, the classifications in the SFR are made by an unselected group of mostly inexperienced classifiers. The results indicate that the fracture classification in a national quality register can be accurate enough to enable the evaluation of fracture treatment in specific groups of fractures.

## Abbreviations

A&E: Accident and emergency department
AO: Arbeitsgemeinschaft für Osteosynthesefragen
CI: Confidence interval
FDR: Fracture and Dislocation Registry
ICD-10: Tenth revision of International Statistical Classification of Diseases and Related Health Problems
OTA: Orthopaedic Trauma Association
PA: Percentage of agreement
SFR: Swedish Fracture Register
